# Assessment of immunogenicity and drug activity in patient sera by flow-induced dispersion analysis

**DOI:** 10.1038/s41598-022-08682-3

**Published:** 2022-03-18

**Authors:** Morten E. Pedersen, Jesper Østergaard, Bente Glintborg, Merete L. Hetland, Henrik Jensen

**Affiliations:** 1 Fida Biosystems ApS, Generatorvej 6, 2860 Soeborg, Denmark; 2 Department of Pharmacy, University of Copenhagen, Universitetsparken 2, 2100 Copenhagen O, Denmark; 3 The DANBIO Registry and Copenhagen Center for Arthritis Research (COPECARE), Center for Rheumatology and Spine Diseases, Centre of Head and Orthopaedics, Copenhagen University Hospital Rigshospitalet, 2600 Glostrup, Denmark; 4 Department of Clinical Medicine, Faculty of Health and Medical Sciences, University of Copenhagen, Copenhagen, Denmark

**Keywords:** Biological techniques, Immunology, Autoimmunity, Prognostic markers

## Abstract

Biopharmaceuticals have revolutionized the treatment of many diseases such as diabetes, cancer, and autoimmune disorders. These complex entities provide unique advantages like high specificity towards their target. Unfortunately, biopharmaceuticals are also prone to elicit undesired immunogenic responses (immunogenicity), compromising treatment efficacy as well as patient safety due to severe adverse effects including life threatening conditions. Current immunogenicity assays are hampered by immobilization procedures, complicated sample pre-treatment, or rely on cell-based methods which all prevent reliable and continuous monitoring of patients. In this work, we present Flow Induced Dispersion Analysis (FIDA) for assessment of immunogenicity and drug activity in serum samples from arthritis patients receiving adalimumab. FIDA is a first principle technique for size-based characterization of biomolecules and their complexes under biologically relevant conditions. The FIDA methodology rely on an absolute and quantitative readout (hydrodynamic radius) thus reducing the need for positive and negative controls. Here, FIDA is applied for evaluating active adalimumab in serum by studying the interaction with its target tumor necrosis factor alpha (TNF-α). We report proof of principle for a quantitative approach for stratifying patients exhibiting presence of neutralizing and non-neutralizing antibodies based on their individual drug activity pattern. Further, it can be applied to any biopharmaceutical having soluble drug targets and it holds potential in a companion diagnostics setting.

## Introduction

Biopharmaceuticals have become an increasingly important class of therapeutics since they provide major benefits in the treatment of many severe diseases such as auto-immune diseases, cancer and diabetes^1–3^. The primary advantages relate to high specificity and effective replacement or substitution of endogenous compounds (e.g., insulin and erythropoietin)^1,2^. However, administration of biopharmaceuticals to patients may induce an unwanted immune response against the treatment, formally known as immunogenicity^4–6^. The consequences of immunogenicity range from no considerable adverse effects to severe side effects and even anaphylaxis, and/or reduced treatment efficacy^4–6^. Biologically, this manifests through the presence of circulating anti-drug-antibodies (ADAs), which can be divided into neutralizing antibodies (NAbs) and non-neutralizing antibodies (non-NAbs)^4–6^.

Analytical assays for detecting and quantifying ADAs, including competitive ligand binding assays, have clinical significance in terms of ensuring patient safety and treatment efficacy as well as guiding choice of treatment regime^4,5,7^. Typically, ADA assays rely on a relative signal readout using cell-based assays or surface-based ligand binding assays (LBAs), such as enzyme-linked immunosorbent assay (ELISA), electrochemiluminescence (ECL), and surface plasmon resonance (SPR), where one of the interaction partners (e.g., the drug) is immobilized on a surface^4,7,8^. These technologies do not resemble the human biology as immobilization procedures can mask relevant epitopes and alter the conformation of key reagents, e.g., the drug^7^. Most assays cannot directly distinguish between the presence of non-NAbs or NAbs, hence requiring multiple assays and orthogonal techniques^7,8^. The presence of therapeutic drug in the sample may also affect assay performance negatively depending on the drug tolerance limit of the assay^8^. Furthermore, most LBAs only report presence of ADAs, and do not provide information regarding the activity of the drug in the sample^9,10^. Cell-based assays may assess both drug activity and presence of neutralizing antibodies^11^, and therefore frequently utilized in immunogenicity studies during clinical trials. However their applicability can be limited by technical challenges and validation procedures^7^.

An ideal methodology would provide a measure of immunogenicity under biorelevant conditions, including assessment of ADA type, i.e., non-NAbs or NAbs, as well as an assessment of drug activity.

FIDA is a first principle analytical approach employed for size-based characterization of biomolecules under native conditions, including protein unfolding^12^, binding affinity (*K*_d_) and complex size^13–16^, as well as biopharmaceutical quantification^13,14,17^. Briefly, FIDA utilizes Taylor dispersion analysis (TDA) for measuring the apparent hydrodynamic radius, *R*_h_, of a selective ligand which is termed the indicator, as it binds to the analyte of interest^13–16^. The *R*_h_ measurements can be utilized for achieving information on molecular size, structure, oligomerization, and complex/binding stoichiometry^16^. Furthermore, FIDA is compatible with crude matrixes such as plasma and fermentation broth^17–19^. A key advantage of FIDA is the first principle nature of the technology which minimize the need for assay calibration as well as positive and negative controls. Recently, FIDA was used for detailed characterization of the tumor necrosis factor alpha (TNF-α)—adalimumab interaction in buffer and human plasma^16^.

Currently, adalimumab is the world’s best-selling drug and used in the treatment of auto-immune diseases, such as rheumatoid arthritis. It is a fully human monoclonal IgG1 antibody specific against the inflammatory cytokine TNF-α^20–23^. Even though, adalimumab is effective, widely used, and structurally fully human, it is, unfortunately, also known for inducing ADA responses in patients^24,25^. ADA prevalence ranges from 28% (n = 272) over a three years period^26^ to 70% (n = 30) over 28 weeks^27^ have been reported.

Here, we show proof of principle that FIDA can be used for characterizing immunogenicity and drug activity in serum samples from patients in steady-state treatment with adalimumab, employing a single assay format. The apparent hydrodynamic radius of the drug target, TNF-α, was selectively measured in a dilution series of 0.01–40% patient serum. Patient samples were stratified into three groups according to their binding pattern of serum adalimumab to TNF-α: 1) samples exhibiting normal drug activity levels and thus unrestricted adalimumab-TNF-α binding, 2) samples demonstrating reduced drug activity and containing predominantly non-neutralizing antibodies, and 3) samples containing predominantly neutralizing antibodies. As the readout in FIDA is absolute size (*R*_h_) and thus inherently quantitative, the developed assay does not require calibration in the form of control antibodies which may have markedly different properties than treatment induced ADAs found in patients. Our findings were compared to a commercially available anti-adalimumab ELISA kit. The presented FIDA approach is widely applicable to drug—target systems composed of a biopharmaceutical and a soluble drug target. The FIDA approach has the potential to find application in pre-clinical and early clinical assessment of new biopharmaceutical modalities as well as for patient stratification and treatment optimization in current therapeutic solutions.

## Results and discussion

### Assessment of immunogenicity and drug activity in patients receiving adalimumab using FIDA

**Figure 1:**
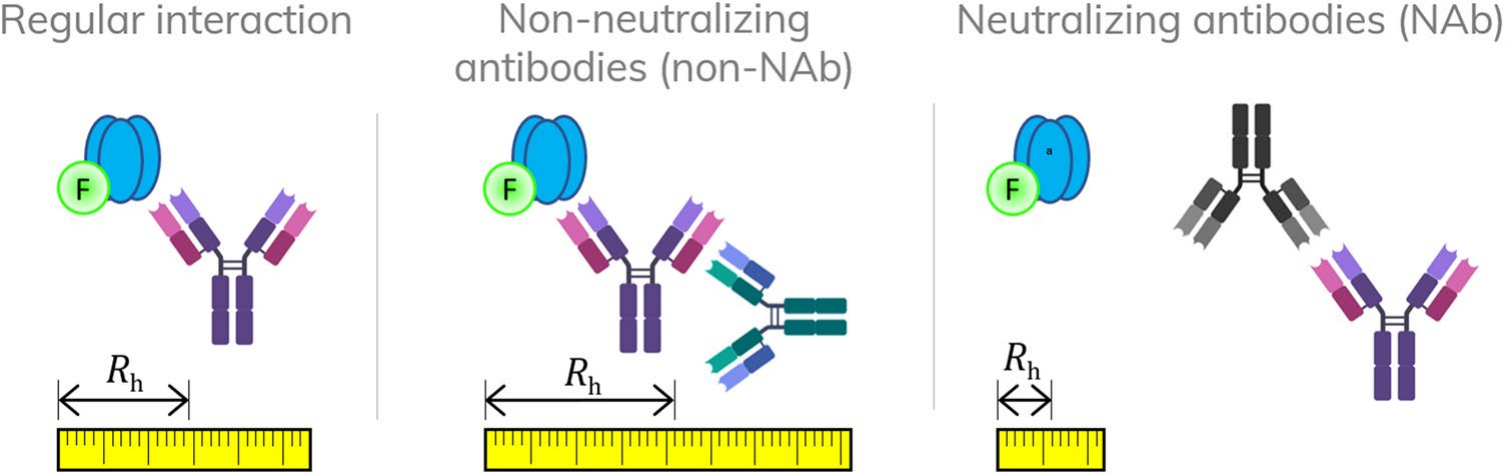
Schematics showing ADA classification as function of apparent hydrodynamic radius (*R*_*h*_) of TNF-α-AF488 (indicator) and adalimumab (analyte). TNF-α-AF488 is illustrated as a soluble homotrimer (blue) conjugated to a fluorophore (green) which enables selective *R*_*h*_ measurements via fluorescence detection. Adalimumab is exemplified as an antibody (bordeaux) targeted by non-neutralizing antibodies (orange) and neutralizing antibodies (black). The yellow ruler illustrates the link between *R*_h_, normal drug activity (left), and presence of ADAs (middle and right). Created by author Morten E. Pedersen using biorender.com.

Characterization of ADAs and active adalimumab in patient serum utilizing FIDA, is based on using the drug target molecule TNF-α as a selective indicator. The apparent size (hydrodynamic radius, *R*_h_) of TNF-α-AF488 is measured in a titration series directly in serum solutions. In case of sufficient free and active adalimumab in serum (i.e., preserved drug activity^9,11^), the apparent size of TNF-α-AF488 increases due to complex formation. Specifically, the size range of the formed complexes can be utilized for assessment of drug activity and immunogenicity in patients receiving adalimumab as indicated in Fig. 1. The TNF-α-AF488 and adalimumab interaction has recently been characterized in human plasma using FIDA^16^. This well-defined dataset forms the basis, and thus benchmark, for anticipated drug activity of adalimumab in human plasma samples^16^. Regular responders (i.e., non-immunogenic) will return *R*_h_ (TNF-α-AF488)-values similar to the benchmark due to normal drug activity level of adalimumab, whereas *R*_h_ (TNF-α-AF488)-values above the benchmark imply the presence of higher order complexes formed between non-neutralizing antibodies, adalimumab and TNF-α. Finally, *R*_h_ (TNF-α-AF488)-values corresponding to free target (unbound TNF-α-AF488) indicates neutralizing antibodies and hence no drug activity, see Fig. 1.

**Figure 2:**
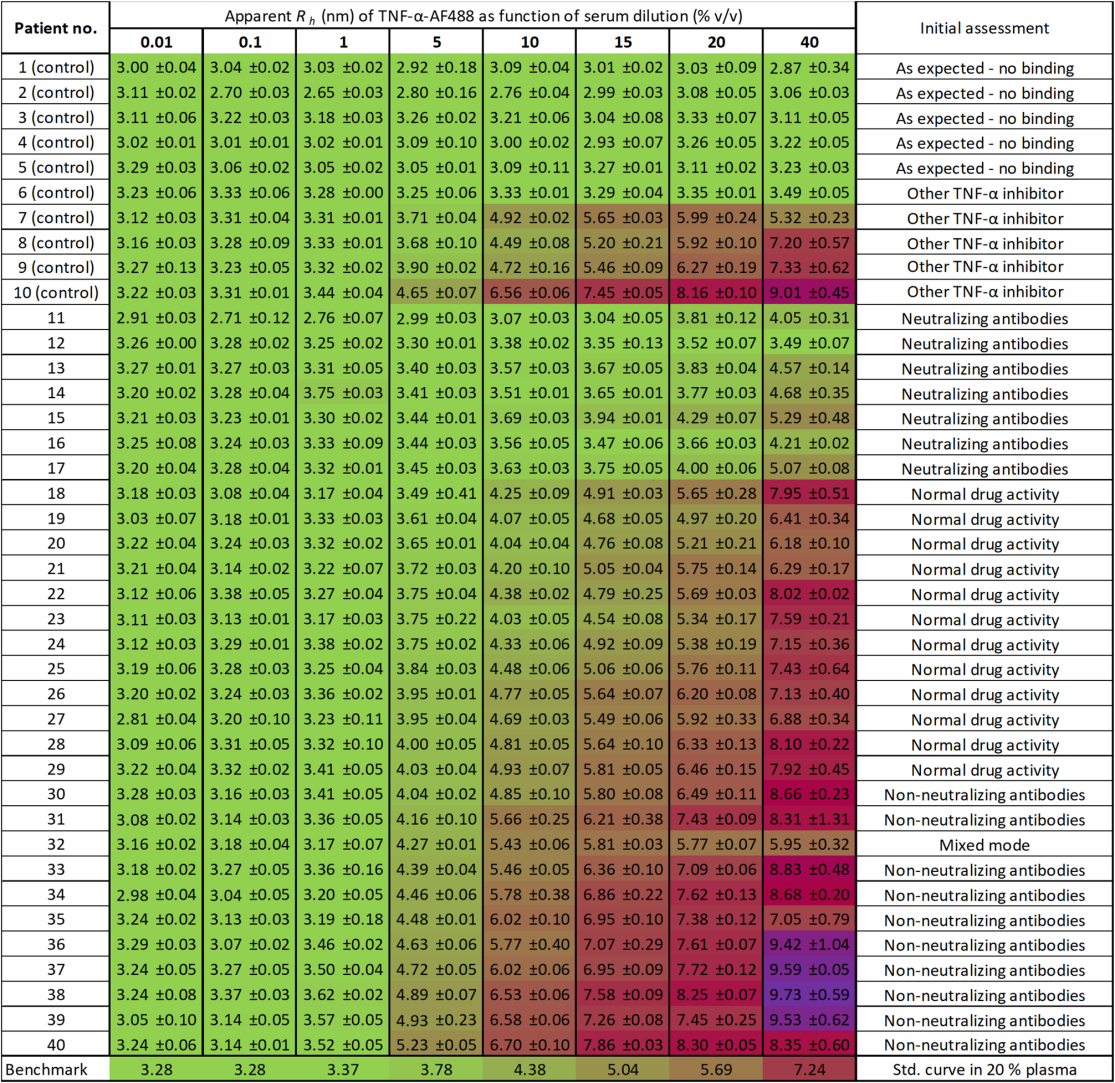
Overview and heatmap of 40 patient samples, showing the apparent hydrodynamic radius of TNF-α-AF488 (100 nM) as function of serum concentration (0–40% v/v) diluted with assay buffer, determined by FIDA at 25 °C (n = 3) with pre-incubated samples (> 10 min). Patients were renumbered and arranged according to response type. Last row depicts benchmark, based on values from a std. curve in 20% v/v plasma.

In practice, the apparent *R*_h_ of the indicator, TNF-α-AF488 (100 nM), was measured in a titration series with patient serum (0.01–40% v/v) diluted with assay buffer (Fig. 2). In the control group, 6 of 10 patients did not show any increase in the apparent size of TNF-α-AF488, thereby, as expected, indicating the absence of an interaction between the indicator (TNF-α-AF488) and human sera components, which is consistent with the absence of adalimumab. Surprisingly, 4 of 10 samples from the control group exhibited TNF-α binding. Subsequently, the sample provider confirmed that patients 6–10 were treated with other TNF-α inhibitors such as etanercept, infliximab, and certolizumab. Despite being in treatment with a TNF-α inhibitor, an increase in hydrodynamic radius and consequently interaction with TNF-α-AF488 was not observed for patient 6. The results from the control group of 10 patients verified that the indicator, TNF-α-AF488, was selective and applicable to other TNF-α inhibitor therapies. Furthermore, the measured complex sizes were indicative of the type of anti-TNF-α therapy administered, since certolizumab pegol (48 kDa) might result in a lower plateau value than adalimumab (148 kDa), which may be the case for patient 7 at 15–40%, where the complex size was plateauing around 5–6 nm (Fig. 2).

The experimental group (patients 11–40) were compared to the binding curve for TNF-α-AF488 and adalimumab in 20% v/v human plasma, which has been previously characterized by FIDA using final drug substance (adalimumab) and the same experimental parameters^16^. The binding curve was used for calculating benchmark values of *R*_h_ (TNF-α-AF488) concerning the anticipated level of active adalimumab in the patients receiving steady-state treatment with 40 mg adalimumab s.c. every other week, see last row in Fig. 2. Details on how to calculate the benchmark *R*_h_ (TNF-α-AF488)-values to each serum concentration can be found in the supplementary information section. The endogenous level of TNF-α is in the pM-range and thus not expected to impact assay performance^28^. Most of the experimental patient group (n = 12, 18–29) exhibited apparent sizes of TNF-α-AF488 similar to the benchmark at different serum dilutions, indicating a regular interaction and normal drug activity of adalimumab in sera.

Interestingly, a relatively large group of the patients (n = 7, 11–17) showed a substantially impaired interaction between the serum adalimumab and TNF-α-AF488, which was apparent as low hydrodynamic radii when compared to the benchmark values and the regular response pattern (18–29) (Fig. 2). This must be interpreted as reduced drug activity due to low concentrations of free and active adalimumab in sera, pointing to presence of circulating neutralizing antibodies directly obstructing the interaction between TNF-α and adalimumab. As an example, patient 12 lead to apparent size measurements corresponding to unbound TNF-α at all dilutions (0–40% v/v serum), implying virtually no adalimumab activity. This was also the case for patient 6 in the control group, which suggested NAbs against another TNF-α inhibitor. In principle, the results can also be rationalized in terms of a substantially reduced level of adalimumab in the patient samples, however, incorrect drug administration is unlikely to have occurred in a significant part of the patients.

### Quantitative ADA classification parameter and cut-off

**Figure 3:**
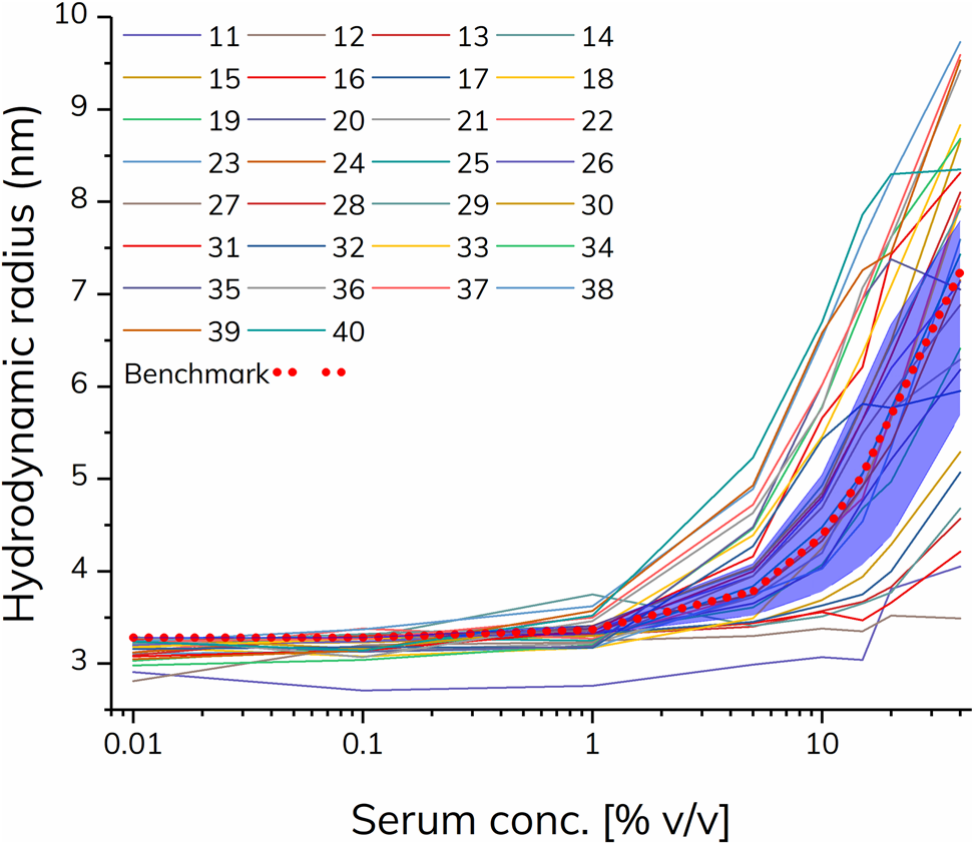
Experimental group (30 patients) depicted as lines with apparent hydrodynamic radius of TNF-α-AF488 (100 nM) as a function of serum concentration (0–40% v/v). The red dotted line shows theoretical benchmark values using the standard curve in 20% v/v plasma. The blue zone demonstrates 50% deviation in steady-state serum concentration of 8 µg/mL (~ 54 nM) adalimumab.

While it was straightforward to probe drug activity of adalimumab and identify NAb positive patients, it may be more challenging to differentiate regular drug activity from non-NAb positives, at least at certain adalimumab concentrations, because certain absolute and relative concentrations of TNF-α and adalimumab may result in the formation of higher structures between trimeric TNF-α and bivalent adalimumab^16^. The developed FIDA assay provided immediate information on target binding and thus assessment of drug activity, however, the structural readout (*R*_*h*_) in FIDA could be utilized for further ADA classification of the patients. The binding offset and pattern for each patient shown in Fig. 3 may be indicative of the ADA subtype, since the presence of NAbs will reduce the drug activity of adalimumab, and thus right shift the binding curve towards higher serum concentrations. On the other hand, the presence of non-NAbs will lead to formation of higher order complexes consisting of adalimumab, non-NAb and TNF-α, which would left shift the binding curve when depicting apparent *R*_h_ of TNF-α *versus* the serum concentration. This approach is visualized in Fig. 3. The blue section is a set region corresponding to regular behavior (i.e., normal responders) and normal drug activity, where NAbs and non-NAbs are absent from the patient serum samples. The cut-off region was tentatively based on a ± 50% deviation in target serum adalimumab concentration of 8 µg/mL, which was similar to variations reported recently^21,29^^.^ As previously described^16^, FIDA is a first principle approach that allows the influence of assays parameters (including drug concentration) on assay response to be quantified. The regions above and below the curve corresponds to patients having developed non-NAbs and NAbs, respectively. The different regions can also be described more quantitatively. Here, we propose to simply use the change in apparent *K*_d_-value, for each patient, as a parameter for describing the effect of NAbs and non-NAbs on a quantitative scale, since this parameter reflects a combination of drug activity, and ADA amount, affinity, and subtype.

The patient-specific apparent *K*_d_-value, here termed drug activity factor (*DAF*), was calculated by fitting the excess binding isotherm (described in supplementary information) to each patient’s dataset (0.01–40% serum) using the benchmark values as fixed parameters, i.e., the *R*_complex_, *R*_indicator_, *I* (diluted indicator concentration), and *K*_d_ (*DAF)* as fitting parameter, see Table 1. A more general approach for data treatment could be to use a normalized drug activity factor (*DAF*_Norm_) as listed in Table 1. The *DAF*_Norm_ is calculated as the ratio of the patient and benchmark *K*_d_ and is thus a dimensionless number. A *DAF*_Norm_ significantly above 1 indicates predominantly non-NAbs whereas a value significantly below 1 indicates the presence of NAbs. Here, the benchmark *DAF* for adalimumab was 3.19% serum and thus applied for normalizing all calculated *DAF* values.

**Table 1:**
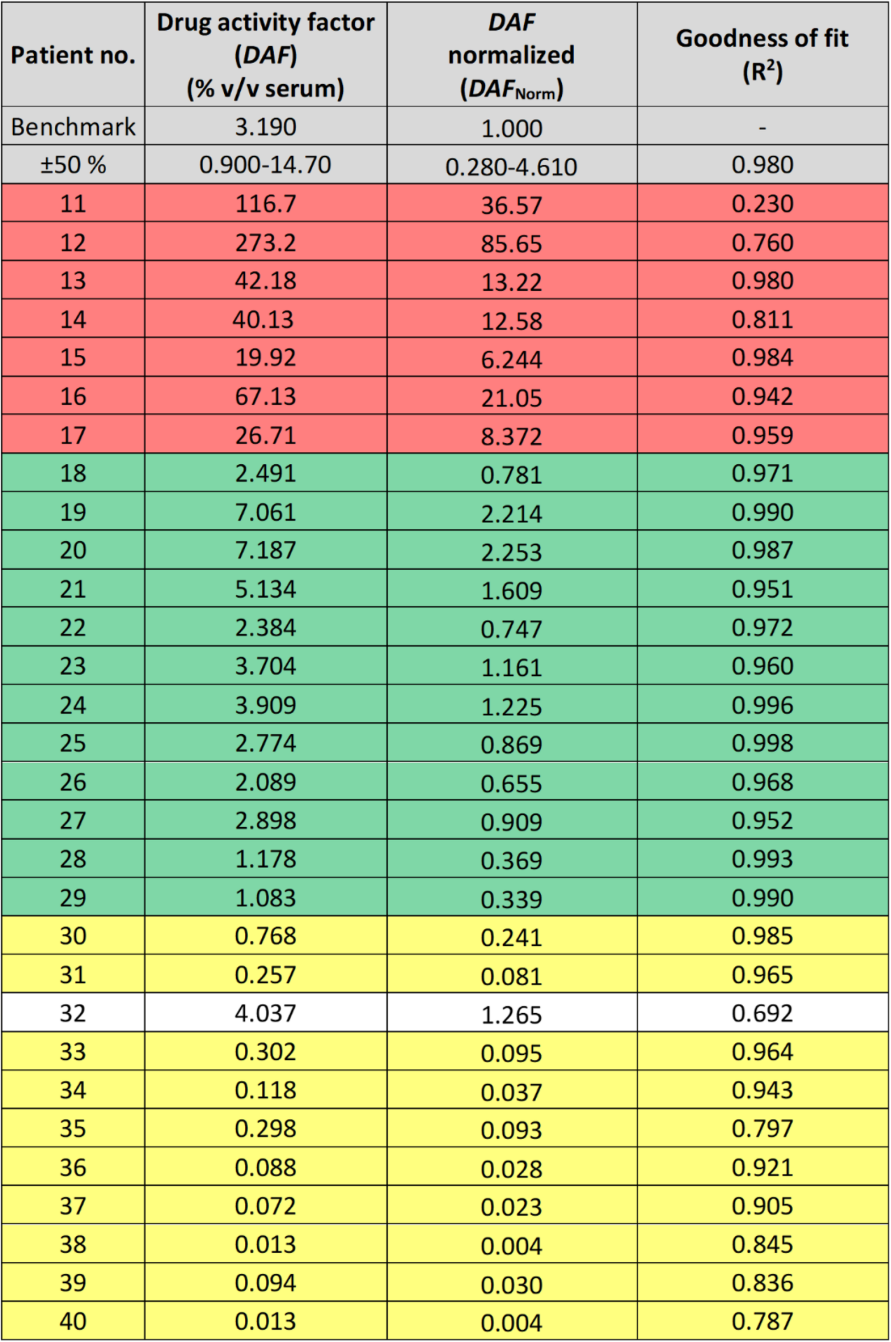
Calculated drug activity factors (DAF) for the experimental group (30 patients), based on fitting to the excess indicator binding isotherm with parameters from the standard curve in 20% plasma [i.e., R_complex_ = 8.71 nm, R_indicator_ = 3.28 nm, and I = 19.40% serum (corresponding to 10.47 nM TNF-α)].

The red, green, and yellow color codes correspond to patients classified as NAb positive, normal responders, and non-NAb positive, respectively. Patient 32 showed signs of mixed mode ADAs, and was therefore not classified.

Regular responders with normal drug activity of adalimumab had DAF_Norm_ values inside range 0.28–4.61. Again, this was based on ± 50% deviation from the reported steady state serum concentration of adalimumab^21,29^. However, it may be further qualified by using a larger data set and including clinical data. Based on these observations, the remaining patients in the experimental group (n = 10, patients 30, 31, 33–40) were classified as non-NAb positive, since they exhibited the following indications; 1) DAF_Norm_ values multiple orders of magnitude below the benchmark value (Table 1), which was visually seen as left shifts in the patient binding curves (Figs. 2 and 3) the majority demonstrated relatively large complex sizes, hence indicating formation of higher order complexes composed of TNF-α, adalimumab and non-NAb. We did observe an outlier, patient 32, which exhibited an apparently regular DAF_Norm_ value (1.265) but had an initial left shifted offset combined with a low plateau value corresponding to both non-NAbs and NAbs, respectively. It was speculated that patient 32 may have a presence of high affinity non-NAbs that bind the TNF-α-adalimumab complex at low serum concentration, however, at higher serum concentrations the presence of low affinity NAbs partially obstruct formation of the TNF-α-adalimumab complex. As the FIDA methodology has an absolute size readout such behavior is straight forward to characterize.

In the present study of 30 patients receiving adalimumab, the developed FIDA assay found 12 (~ 40%) patients with normal drug activity, 7 (~ 23%) patients with presence of neutralizing antibodies, and 10 (~ 33%) patients with indications of non-neutralizing antibodies, and finally 1 (~ 4%) patient with mixed mode. The 40 patient samples were also analyzed using a commercially available, EMA and FDA validated, anti-adalimumab ELISA kit. However, the ELISA results indicate that only one patient (patient 24) had developed anti-adalimumab antibodies during adalimumab treatment lasting for at least 12 months, thus reporting an ADA positive percentage of 3.3%. These findings were directly contradictive to the results obtained with the FIDA assay, as well as other studies in the literature showing ADAs in 28%^26^ and 70%^27^ of the patients. The underestimation of ADAs was likely caused by drug interference from adalimumab present in the serum samples, where the assay reagents (i.e., plate-bound adalimumab and adalimumab-HRP) will be in competition with adalimumab in serum as well as complex-bound anti-adalimumab antibodies. These challenges have previously been addressed by introduction of acid dissociation steps to improve assay sensitivity^30,31^. It should be noted that a recent study found a pronounced discrepancy between SPR and ELISA based assays, presumably due to antibodies not being detected in ELISA due to fast off rates in the capture step^32^. This underlines the complexity in comparing different methodologies for ADA testing thus pointing to the use of clinical data as the most informative route for validating the assay.

The neutralizing capacity of the patient population having anti-adalimumab antibodies (total NAbs/(total NAbs + non-NAbs)) was 41% (i.e., 7/17) in the developed FIDA assay. This result was similar to a study by Hock et al., reporting neutralizing capacities in a patient population of 48–54% for anti-adalimumab antibodies, using homogenous mobility shift and affinity capture elution assays followed by ELISA^33^. However, a number of studies have reported neutralizing capacities of up to 97–98% for the entire anti-adalimumab population^34,35^. However, these studies were primarily based on high ADA level responders where low ADA level patients were excluded, as well as immobilization methodologies^35^. A key advantage of using an in-solution assay is that the weak binding ADAs are also measured in contrast to surface-based technologies with multiple wash steps, which may eliminate low affinity antibodies.

It is emphasized that the developed FIDA assay is not limited to a ligand binding assay probing target binding and thus drug activity. The structural readout in form of *R*_*h*_ provides important information regarding the interaction and can be used for classifying neutralizing and non-neutralizing antibodies in combination with a benchmark. The relatively low complex sizes for the NAb positive patients may be ascribed to higher clearance rates for adalimumab induced by any type of ADA^36,37^^.^

### Generalized data treatment

**Figure 4:**
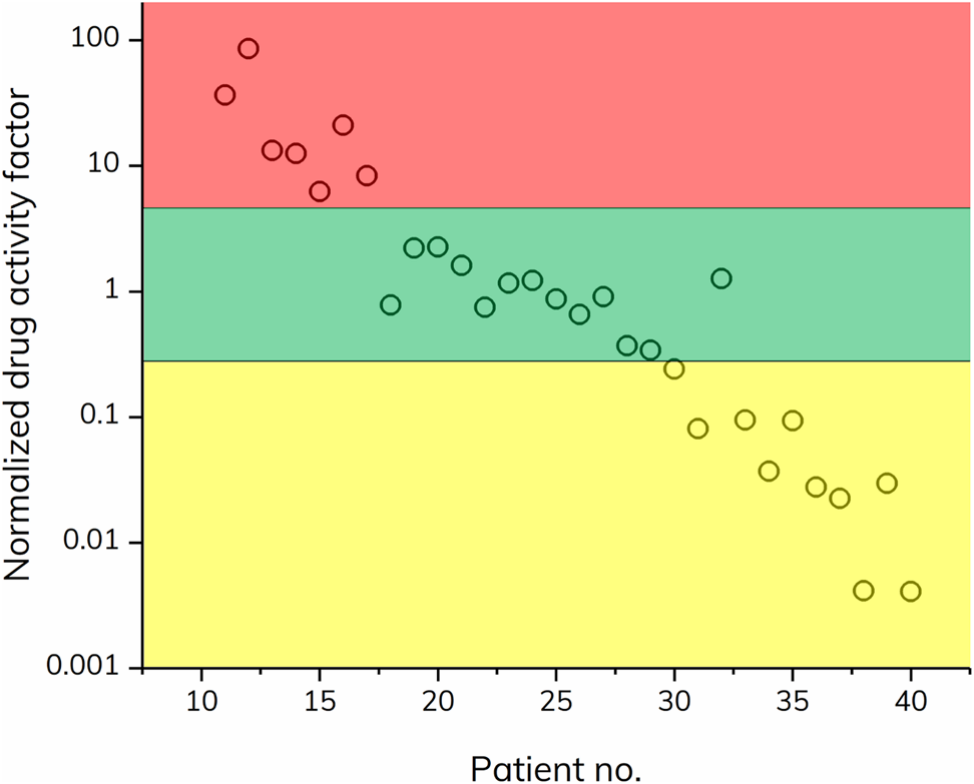
Visual representation of normalized drug activity factor (DAF_Norm_) for as patient number 11–40 from the experimental group (30 patients). Again, the red, green, and yellow color zones correspond to patients classified as NAb positive, normal responders, and non-NAb positive, respectively.

The cut-off for different drug response classifications are dependent on the specific biopharmaceutical drug and should be determined using combined knowledge of intra-patient drug levels variability and clinical data. *DAF*_Norm_ can be visualized in a given patient population as shown in Fig. 4, which is a visual representation of the patient population and thus allows for rapid comparison of different biopharmaceutical drugs and their propensity for inducing unwanted immunogenicity. In addition to following a patient population, a similar diagram with *DAF*_Norm_ *versus* time may be used to follow individual patients allowing treatment adaption prior to occurrence of adverse effects.

This approach can be applied for most biopharmaceutical drugs and can be used for linking drug activity and immunogenicity to treatment strategies such as choice of modalities, doses, formulations, and route of administration.

## Conclusions

Proof of principle of a new methodology has been developed which enable simultaneous assessment of drug activity and immunogenicity of biopharmaceuticals in human serum. The methodology was applied to serum samples from 40 arthritis patients treated in routine care. The size of TNF-α-AF488 was selectively measured in a titration series with patient sera, thereby obtaining the apparent complex sizes between TNF-α-AF488 and adalimumab present at different serum dilutions. The first principle FIDA assay measured TNF-α complex sizes, thus the size ranges measured for each patient could be utilized for assessment of free and active adalimumab as well as complex-bound adalimumab caused by the presence of neutralizing or non-neutralizing antibodies. The patient response type and ADA subtype were determined quantitatively from the calculated patient-specific *K*_d_-value (termed *DAF*), which may be used as a parameter in the clinic for evaluating adalimumab treatment strategy.

A new parameter is suggested (*DAF*_Norm_) which may be generally applicable for characterizing immunogenicity in patients receiving treatment based on biopharmaceuticals. The *DAF*_Norm_ cut-off values may be based on clinical manifestations as well as independent orthogonal methodologies.

The developed FIDA methodology is a promising tool for assessment of both immunogenicity and drug activity in biopharmaceutical therapies ultimately leading to better therapies with reduced side effects. Advantages compared to existing technologies used for identifying and characterizing the formation of ADAs include concurrent information on both immunogenicity and drug activity in a single assay as well as biorelevant assay conditions. A key feature of the FIDA methodology is that it lends itself to miniaturization enabling feature devices for decentralized companion diagnostics. Further development includes other drug modalities and soluble targets with lower therapeutic dose as well as direct linkage to clinical observations.

## Methods

### Materials

#### Patient samples

Forty patient samples were provided by the Danish Rheumatologic Biobank (Rigshospitalet Glostrup) consisting of sera from two groups: experimental (30 samples) and control (10 samples). Samples in both groups were from patients diagnosed with rheumatoid arthritis, psoriatic arthritis or axial spondylarthritis as recorded in the DANBIO registry^38^. Patients providing samples for the experimental group were in steady-state monotherapy with adalimumab (40 mg s.c. every other week) for at least 12 months at the sampling time, whereas the control group had never received adalimumab therapy. However, retrospectively it was discovered that five members of the control group were in ongoing treatment with other anti-TNF-α therapies (e.g., infliximab, etanercept, or certolizumab pegol). Approval was given by the Danish Ethical Committee system and Data Protection Agency. The serum samples were collected following oral and written informed consent (Biomarkerprotocol^39^), according to the guidelines by the Danish Ethical Committee system and Data Protection Agency (Act on Research Ethics Review of Health Research Projects). All data was Pseudonymization, and patients were renumbered from their original sample number and arranged according to response type.

#### Chemicals and reagents

Recombinant human TNF-α (cat. no. ab155699) was purchased from Abcam (Cambridge, United Kingdom). Alexa Fluor 488 (cat. no. A20181) labeling kits were purchased from Thermo Fisher (Waltham, MA, USA). TNF-α was labelled with Alexa Fluor 488 as described previously^16^. Anti-adalimumab ELISA kit (cat. no. KBI2015, V 2.1) was purchased from Eagle Biosciences (Amherst, NH, USA). Disodium hydrogen phosphate dihydrate, and sodium dihydrogen phosphate monohydrate were purchased from Merck (Darmstadt, Germany). Purified water (18.2 MΩ-cm, 25 °C) was obtained from an SG Ultraclear water purification system (SG Water, Barsbüttel, Germany).

A 67 mM phosphate buffer (pH 7.4) was prepared, filtered (Q-max 0.45 µm nylon syringe filter from Frisenette, Knebel, Denmark) and used as assay buffer.

### Equipment

FIDA experiments were conducted on a Fida 1 instrument employing light-emitting-diode (LED) induced fluorescence detection with an excitation wavelength of 480 nm and emission wavelengths > 515 nm from Fida Biosystems ApS (Copenhagen, Denmark). Standard capillaries with inner diameter 75 µm, outer diameter 375 µm, total length 100 cm, length to detection window 84 cm, from Fida Biosystems were used for all experiments. The capillaries were coated with high-sensitivity (HS) coating reagent (Fida Biosystems) prior to use.

### Methods

#### FIDA procedure

The following stepwise procedure was applied for the FIDA experiments with an overall analysis time of 5–6 min per sample. First, assay buffer (pH 7.4; 67 mM) was used for flushing and equilibrating the HS-coated capillary at 3500 mbar for 120 s. The analyte sample (diluted patient serum) was then filled into the capillary at 3500 mbar for 20–25 s followed by injection of the indicator (TNF-α-AF488 in patient serum) at 50 mbar for 10 s. Lastly, the indicator was mobilized to the fluorescence detector with analyte sample (diluted patient serum) at 400 mbar for 180–240 s depending on viscosity. The taylorgram was recorded in the latter step. The same procedure can also be used for human plasma samples^16^.

#### Characterization of adalimumab immunogenicity in patient sera by FIDA

The indicator, TNF-α- AF488, concentration was fixed at 100 nM and titrated with patient serum (0.01–40% v/v) diluted with assay buffer. All samples were preincubated for > 10 min prior to the FIDA measurements and measured in-triplicate.

#### ELISA procedure

The patient samples were analyzed for the presence of anti-adalimumab antibodies using a commercially available sandwich-ELISA kit. Each patient sample was analyzed at two dilutions (1:10 and 1:100), following the vendor’s instructions. The standard curve was established in duplicates, at 0–320 ng/mL (i.e., 0–2.1 nM), using the provided anti-adalimumab antibody standard.

### Data analysis

The raw data (i.e., the taylorgrams) were analyzed using FIDA software (V 2.11, Fida Biosystems ApS, Copenhagen, Denmark) to determine the apparent hydrodynamic radius of TNF-α- AF488 at each patient serum concentration. The fitting was set to standard (i.e., 75% Taylorgram fraction) for all data points. Patient serum viscosity was calculated by the software, and automatically applied for correction of the apparent hydrodynamic radius to a reference measurement in neat assay buffer at 25 °C, as previously described^12^. The principles for calculating the hydrodynamic radius from the taylorgrams have been described elsewhere^17^.

## Supplementary Information

Supplementary Figures.

## Data Availability

The raw data (i.e., taylorgrams) and data analysis are available upon request to the corresponding author.
