# Supplementary material for: Assessment of immunogenicity and drug activity in patient sera by flow-induced dispersion analysis
Source: Sci Rep. 2022 Mar 18;12:4670. doi: 10.1038/s41598-022-08682-3 (PMC8933425; doi:10.1038/s41598-022-08682-3)
Supplement: Supplementary file 1 — Supplementary Figures. [file 41598_2022_8682_MOESM1_ESM.docx]

**Supplementary information**

**Assessment of Immunogenicity and Drug Activity in Patient sera by Flow-Induced Dispersion Analysis**

Morten E. Pedersen^a,b^, Jesper Østergaard^b^ , Bente Glintborg^c,d^, Merete L. Hetland^c,d^, and Henrik Jensen^a^

*^a^ Fida Biosystems ApS, Generatorvej 6, 2860 Soeborg, Denmark.*

*^b^ Department of Pharmacy, University of Copenhagen, Universitetsparken 2, 2100 Copenhagen O, Denmark.*

*^c^* *The DANBIO registry and Copenhagen Center for Arthritis Research (COPECARE), Center for Rheumatology and Spine Diseases, Centre of Head and Orthopaedics, Copenhagen University Hospital Rigshospitalet, 2600 Glostrup, Denmark*

*^d^Department of Clinical Medicine, University of Copenhagen, Faculty of Health and Medical Sciences, Copenhagen, Denmark*

**Benchmark values for apparent hydrodynamic radius of TNF-α-AF488 as function of active adalimumab**

The experimental patient group (30 individuals) received a subcutaneous dose of 40 mg adalimumab every other week, equivalent to an expected a steady state serum concentration of 8 µg/mL^1^, corresponding to an adalimumab concentration of 54 nM in undiluted serum. Based on this, expected adalimumab concentrations in 0.01 % - 40 % serum were calculated (Table S1). The TNF-α-AF488-adalimumab interaction was characterized by FIDA in 20 % human plasma^2^. This dataset comprised the benchmark for the anticipated drug activity of adalimumab in human plasma samples and followed the excess indicator binding isotherm^3^.

$$R_{\mathrm{app}}=\left( \begin{aligned} \left( R_{complex} \right)^{-1}\cdot\left( \frac{\left( C_{I}+C_{A}+K_{d} \right)-\sqrt{\left( C_{I}+C_{A}+K_{d} \right)^{2}-4\cdot C_{A}\cdot C_{I}})}{2\cdot C_{I}} \right) \\ +\left( R_{\mathrm{indicator}} \right)^{-1}\cdot\left( \frac{\left( C_{I}-C_{A}-K_{d} \right)+\sqrt{\left( C_{I}+C_{A}+K_{d} \right)^{2}-4\cdot C_{A}\cdot C_{I}})}{2\cdot C_{I}} \right) \end{aligned} \right)^{-1}$$

where *R*_app_, *R*_complex_, and *R*_indicator_ are the apparent, complex, and indicator hydrodynamic radii, respectively, *C*_I_ and *C*_A_ are the formal concentrations of the indicator and analyte, respectively, and *K*_d_ is the dissociation constant.

The fitting parameters obtained in 20 % plasma^2^ and depicted in Fig. S1 were *R*_complex_ (8.70532 nm), *R*_indicator_ (3.27503 nm), *C_I_* (10.47 nM), and *K*_d_ (1.72932 nM). Hence, the apparent hydrodynamic radius of TNF-α-AF488 (*R*_app_) was predicted by applying these parameters and the expected adalimumab concentration corresponding to *C_A_* (Table S1). The binding curve (Fig. S1) exhibit regions with increased uncertainty on *R*_h_ (TNF-α-AF488), approximately from 30 – 1000 nM due to formation of higher order stoichiometries between trivalent TNF-α and bivalent adalimumab, as described in reference^2^. For this particular interaction, it is thus important to have a series of serum dilutions to follow the distinct binding pattern in a patient sample, and not just a single value. For this reason, eight dilutions were analyzed for each patient between 0.01 – 40 % serum.

Table S1: Expected apparent complex sizes (*R*_h_) of TNF-α-AF488 as function of adalimumab to each serum concentration in patients receiving 40 mg adalimumab s.c. every other week.

| Serum conc. (% v/v) | 0.01 | 0.1 | 1 | 5 | 10 | 15 | 20 | 40 |
| --- | --- | --- | --- | --- | --- | --- | --- | --- |
| Expected conc. (nM) | 0.0054 | 0.054 | 0.54 | 2.7 | 5.4 | 8.1 | 10.8 | 21.6 |
| Expected apparent *R*_h_ of TNF-α-AF488 (nm) | ~ 3.28* | ~ 3.28* | ~ 3.37* | 3.78 | 4.38 | 5.04 | 5.69 | 7.24 |

*Size of unbound TNF-α-AF488





*Figure S1: Apparent hydrodynamic radius of 100 nM TNF-α-AF488 as function of 0-1000 nM adalimumab in 20% v/v human plasma, determined by FIDA in reference^2^ at 25 °C (n = 3). The solid red line represent fitting to the so-called excess indicator binding isotherm with R^2^ of 0.979. Fitting parameters were: R*_complex_ (8.70532 nm), *R*_indicator_ (3.27503 nm), *C_I_* (10.47 nM), and *K*_d_ (1.72932 nM). *Modified with permission from reference^2^.*

**Assessment of immunogenicity responses in patients receiving adalimumab therapy using a commercial ELISA kit**

The 40 patient samples were analyzed with an anti-adalimumab ELISA kit (Krishgen, Eagle Biosciences cat. no. KBI2015, V 2.1). The kit was based on sandwich-ELISA, where adalimumab was pre-coated onto the plate wells, and adalimumab-horseradish peroxidase (HRP) was used for detection of anti-adalimumab antibodies with color conversion of 3,3',5,5'-Tetramethylbenzidine (TMB) substrate. The patient samples were analyzed at two dilutions (1:10 and 1:100) as recommended by the supplier.


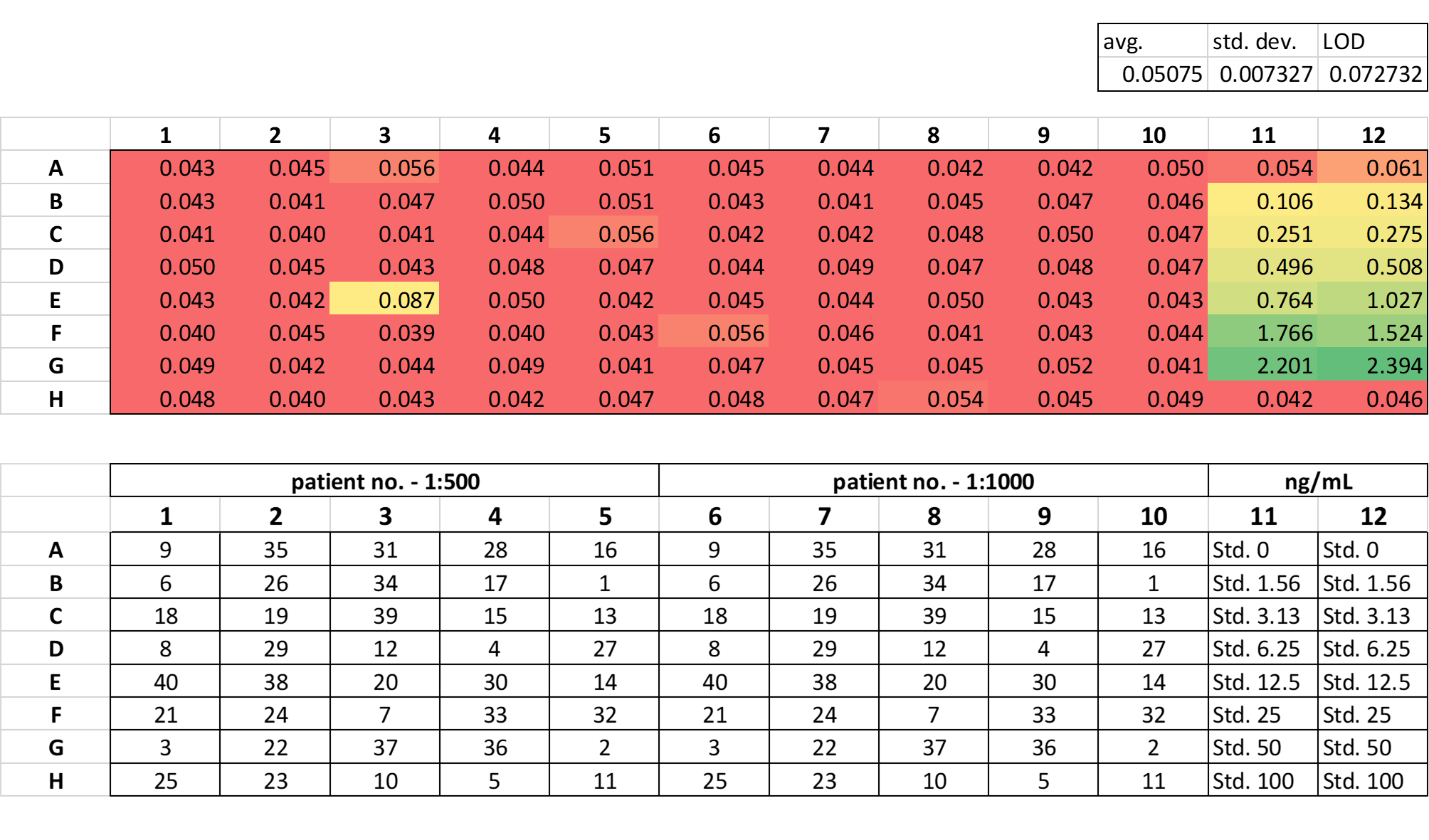


*Figure S2: Overview of 40 patient samples, showing the absorbance values obtained with sandwich-ELISA in 1 – 10 % v/v diluted serum. Positions 1-5 correspond to 10 % v/v patient sera, and position 6-10 is 1 % v/v patient sera, and position 11-12 is the supplier-provided standard curve from 0-320 ng anti-adalimumab/mL. LOD was calculated to 0.073, based on the four measurements in pure buffer (pos. 11A,12A, 11H, and 12H).*

Surprisingly, only one patient (patient 20) exhibited an absorbance value (0.087) above the Limit of Detection (LOD) of 0.073 (Fig. S2). However, it was still below the lowest absorbance values of the ELISA standard curve (0.106 – 0.134) at 10 ng/mL (Fig. S2), hence not applicable for quantification via the established standard curve (Fig. S3). The ELISA results indicate that only one patient (patient 20 had developed anti-adalimumab antibodies during adalimumab treatment for at least 12 months, thus reporting an ADA positive percentage of 3.34 %.





*Figure S3: Standard curve obtained with commercially available anti-adalimumab ELISA kit from 0-320 ng/mL (anti-adalimumab) measured in-duplicates. Absorbance was measured at 450 nm.*

**References**

1. Abbvie. Humira Product monograph. 174 (2019).
2. Pedersen, M. E., Haegebaert, R. M. S., Østergaard, J. & Jensen, H. Size-based characterization of adalimumab and TNF-α interactions using flow induced dispersion analysis: assessment of avidity-stabilized multiple bound species. *Sci. Rep.* 11, 1–10 (2021).
3. Pedersen, M. E., Østergaard, J. & Jensen, H. In-Solution IgG Titer Determination in Fermentation Broth Using Affibodies and Flow-Induced Dispersion Analysis. ACS Omega 5, 10519–10524 (2020).
